# Comparative Viral Sampling in the Sinonasal Passages; Different Viruses at Different Sites

**DOI:** 10.3389/fcimb.2018.00334

**Published:** 2018-09-19

**Authors:** Rachel K. Goggin, Catherine A. Bennett, Ahmed Bassiouni, Seweryn Bialasiewicz, Sarah Vreugde, Peter-John Wormald, Alkis J. Psaltis

**Affiliations:** ^1^Department of Surgery - Otolaryngology, University of Adelaide, Adelaide, SA, Australia; ^2^Queensland Paediatric Infectious Diseases Laboratory, Children's Health Queensland, Brisbane, QLD, Australia; ^3^Child Health Research Centre, The University of Queensland, Brisbane, QLD, Australia

**Keywords:** microbiome, sampling, sinus, sinusitis, virus, virome

## Abstract

**Background:** With the emergence of the microbiome as an important factor in health and disease in the respiratory tract standardised, validated techniques are required for its accurate characterisation. No standardised technique has been reported specifically for viral sampling in the sinonasal passages.

**Aim:** To optimise viral sampling techniques from the sinonasal cavity.

**Methods:** Sterile cytology brushes were used under endoscopic guidance to sample the sinonasal mucosa at time of endoscopic sinus surgery at both the middle and inferior meatuses (MM and IM). DNA and RNA were extracted from the samples and underwent PCR or RT-PCR testing, respectively, for a panel of 15 common upper respiratory tract viruses.

**Results:** Twenty-four adult patients were recruited for this study. 18/24 (75%) patients were positive for virus in at least one site, while 8/24 (33%) were positive for virus at both sites. The mean number of viruses identified at the two sites were similar (0.875 ± 0.899 at the MM vs. 0.750 ± 1.032 at the IM). 6/24 (25%) of patients showed no virus at either site, while 3/24 (12.5%) demonstrated the same viral species at both sites.

**Conclusion:** Although the number of viruses present at different sites with the nasal cavity are similar, discord exists in the viral species between sites. It is therefore recommended that both sites are sampled in the clinical and research setting better to characterise the viral species within the nasal cavity.

## Introduction

The role of the healthy human microbiome in prevention and eradication of disease is an area of burgeoning interest in recent years. The interplay between various colonising organisms, their relative abundance, and the importance of a fine microbial balance has been shown to be essential for normal functioning of multiple organ systems, not least respiratory (Lloyd-Price et al., 2016; Mitchell and Glanville, 2018). Conversely, disruption of this balance between viruses, bacteria, and single-celled eukaryotes has been implicated in numerous disease processes, including acute infective processes as well as many chronic inflammatory diseases (Lloyd-Price et al., 2016).

Microbial dysbiosis (specifically bacterial) has been implicated in several respiratory diseases, including asthma (Fazlollahi et al., 2018) and chronic rhinosinusitis (CRS) (Cleland et al., 2016). Persistent nasal and paranasal sinus inflammation characteristic of CRS affects up to 16% of the western population (Fokkens et al., 2007) and manifests as nasal congestion, facial pain or pressure, anterior or post-nasal drainage, and reduction or loss of smell (Benninger et al., 2003). Although the exact aetiopathogenesis of this condition remains elusive, it is considered multifactorial in origin. Current theories include the fungal hypothesis, the bacterial hypothesis (implicating dysbiosis with Staphylococcus aureus overgrowth, superantigen production, and biofilm formation), and an overactive immune response (resulting in chronic inflammation and defective mechanical and innate immune barriers to infection in the CRS population) (Lam et al., 2015). An area that is anecdotally suggested to play a role in CRS pathogenesis is a viral dysbiosis (Jang et al., 2006; Cho et al., 2013). This is due to self-reports by many CRS patients that their symptoms almost invariably developed after an initial viral upper respiratory tract infection (URTI). Research into the ideal method to sample the sinonasal bacterial microbiome is ongoing (Copeland et al., 2018), however similar efforts to investigate and standardise sampling of the virome have not been made.

Studies attempting to investigate the upper respiratory virome are limited. The lack of standardisation in sampling has led to conflicting results regarding the presence of virus and the composition of the virome. Collection techniques employed thus far include nasal washes, aspirates, brushings, and traditional viral swabs, with viral analysis performed by PCR (Cheung et al., 1993; Tao et al., 1995, 1996; Ramadan et al., 1997; Jang et al., 2006; Zaravinos et al., 2009; Wood et al., 2011; Cho et al., 2013; Costa et al., 2014; Liao et al., 2014; Abshirini et al., 2015; Lima et al., 2015; Rowan et al., 2015). Few studies have compared sampling methods; Heikkinen et al. found no difference in the detection of childhood influenza comparing nasal swabs and aspirates (Heikkinen et al., 2002). Spyridaki et al. found a higher detection of rhinovirus (RV) in nasal lavages compared with nasal brushings, but found no difference in any other viruses tested when comparing these to nasal aspirates and swabs (Spyridaki et al., 2009). To date there have been no studies that have compared different sites within the sinuses and nasopharynx in terms of viral detection.

The aim of the study here presented was to establish differences in viral detection and species sampled from different sinonasal sites, in an effort to validate and standardise viral collection techniques, and facilitate further investigation of the sinonasal virome.

## Materials and methods

### Study participants

Patients for this study were recruited from the tertiary rhinologic practices of the two senior authors (PJW and AJP). This study was carried out in accordance with the recommendations of the Central Adelaide Local Health Network Ethics Committee (HREC/15/TQEH/132). The protocol was approved by the same. All subjects gave written informed consent in accordance with the Declaration of Helsinki. Patients were included in this study if they were older than 18 years of age and were undergoing endoscopic surgery. Control patients consisted of patients with an absence of clinical or radiologic evidence of CRS. CRS patients fulfilled the diagnostic criteria for CRS as outlined in the American guidelines (Rosenfeld et al., 2015). The radiological severity of disease was scored for all patients using a Lund-Mackay score (LMS) (Lund and Mackay, 1993).

### Sampling and processing

**Figure 1:**
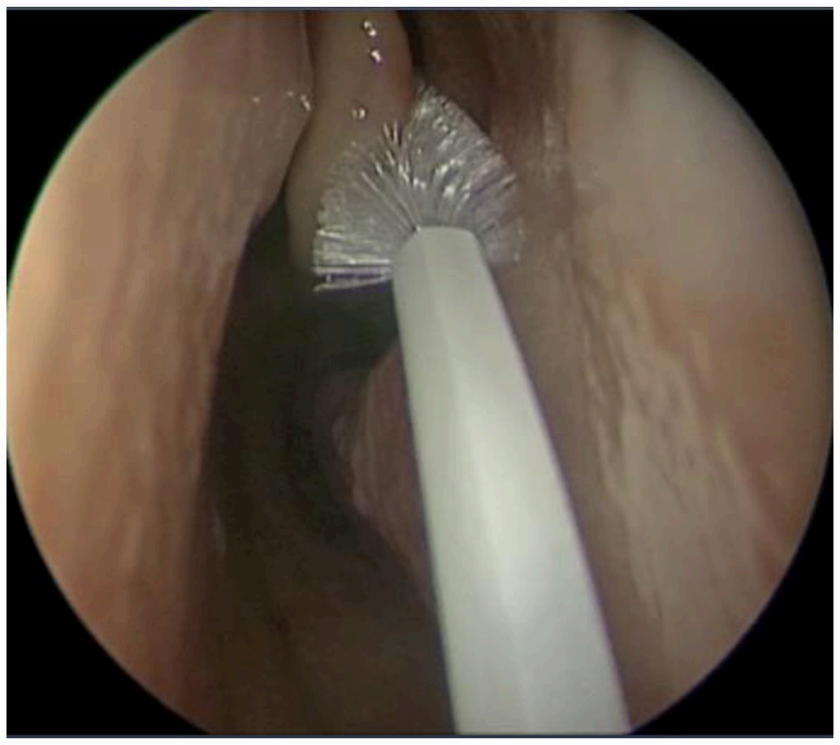
Cytology brushing of sinonasal mucosa.

Using an aseptic technique, endoscan cytology brushes (McFarlane Medical, Melbourne, Australia) were used to sample the sinonasal mucosa (Figure 1) of the left and right middle meatuses (MM) and inferior meatuses (IM) of each patient. This was done under endoscopic visualisation with caution to avoid cross-contamination from neighbouring tissue. The samples were then placed in a viral transport medium [89% Roswell Park Memorial Institute medium supplemented with 9% foetal bovine serum, 1% amphotericin B, and 1% penicillin streptomycin (all Gibco by ThermoFisher, Waltham, USA)] and immediately transported on ice to the laboratory for processing. Sample material was removed from the brushes and centrifuged at 4°C and 1,700 rpm for 7 min in order to isolate cellular material. The supernatant was discarded, after which samples were stabilised with 35 μL RPE Buffer (Qiagen, Hilden, Germany) and 3.5 μL beta-mercaptoethanol (Gibco by ThermoFisher, Waltham, USA) and stored at −80°C.

Samples were thawed in batches to undergo RNA and DNA extraction using an AllPrep DNA/RNA Mini Kit (Qiagen, Hilden, Germany). This yielded DNA samples of 200 μL (average concentration 96.37 ng/μL, range 10.3–383.3 ng/μL) and RNA samples of 60 μL (average concentration 58.58 ng/μL, range 4–247.3 ng/μL).

### PCR/RT-PCR

Extracted DNA and RNA were stored at −80°C until batch testing for a range of upper respiratory tract viruses using real-time PCR. The panel included RV, influenza A–C, parainfluenza (PIV) 1–4, respiratory syncytial virus (RSV) A and B, coronavirus (CoV) HKU-1, OC43, NL63, and 229E, enterovirus (EnV), metapneumovirus (hMPV), adenovirus (AdV), bocavirus (BoV), polyomaviruses WUPyV and KIPyV, Epstein-Barr virus (EBV), cytomegalovirus (CMV), herpes virus 6 (HHV6), herpes simplex virus (HSV) 1 and 2, and varicella zoster virus (VZV). All DNA extracts first underwent an endogenous retrovirus 3 (ERV3) assay (present as two copies per human diploid cell) in order to confirm respiratory sample collection quality.

Briefly, DNA extracts were screened for ERV3, AdV, BoV, WUPyV, KIPyV, CMV, EBV, VZV, HSV 1 and 2, and HHV6 using an identical set of conditions previously optimised so as not to compromise sensitivity (Table 1). Said conditions were 8 pmol of each primer, 3.2 pmol of the respective probe(s), and 2 μL of template, made up to a final reaction volume of 20 μL using the Bioline Sensi Mix II Probe PCR mix kit (Bioline Australia). Details of the target genes, primer, and probe sequences are summarised in Tables 1, 2. Samples then underwent the following cycling conditions: 94°C for 2 min, followed by 45 cycles of 95°C for 15 s and 60°C for 60 s. The RNA extracts were tested for RV, influenza A–C, PIV 1–4, RSV A and B, CoVs HKU-1, NL63, OC43, and 229E, EnV, and hMPV (Table 2) using identical quantities of primer, probe, and template to the DNA reactions but with the Bioline SensiFAST Probe One-Step RT-PCR kit (Bioline, Sydney, Australia). There were two exceptions to these quantities; the IV A/B duplex where asymmetric probe amounts were used (6.4 and 3.2 pmol, respectively) and the RV assay where 16 pmol of probe was used. Samples then underwent the following cycling conditions: 45°C for 20 min, and 45 cycles of 95°C for 15 s and 60°C for 60 s. All samples were run with both positive and negative controls; the positive controls were either previously established clinically positive samples, or synthetic controls specific for each assay. All cycling was conducted on Viia7 instruments (ThermoFisher, Scoresby, Australia). Viral detection was defined as a cycle threshold (Ct) of forty or less.

**Table 1:** Target gene, probe and primer details for DNA viruses.

**Reaction mix**	**Virus**	**Target gene**	**Primer, probe sequences (5^′^-3^′^)**	**Source**
11	Adenovirus	Hexon	GCCACGGTGGGGTTTCTAAACTT GCCCCAGTGGTCTTACATGCACATC FAM-TGCACCAGACCCGGGCTCAGGTACTCCGA-BHQ1	Heim et al., 2003
12	Polyomavirus WU	NCCR	GCCGACAGCCGTTGGATATA TTTCAGGCACAGCAAGCAAT FAM-AGGGTCACCATTTTTATTTCAGATGGGCA-BHQ1	Antonsson et al., 2012
	Polyomavirus KI	NCCR	GAACTTCTACTGTCCTTGACACAGGTA GGATTAGAACTTACAGTCTTAGCATTTCAG Q670-ACCCTTTGTAGGCCAAAGGAGAGTGAAGG-BHQ2	
	Polyomavirus KI	STAg	CACAGGTGGTTTTCTATAAATTTTGTACTT GAAGCAGTGGGATGTATGCATTC YAK-TGCATTGGCATTCGTGATTGTAGCCA-BBQ	
13	Bocavirus	VP1	GGCAGAATTCAGCCATACTCAAA TCTGGGTTAGTGCAAACCATGA FAM-AGAGTAGGACCACAGTCATCAGACACTGCTCC-BHQ1	Tozer et al., 2009
14	Cytomegalo-virus	MIE	AACTCAGCCTTCCCTAAGACCA GGGAGCACTGAGGCAAGTTC FAM-CAATGGCTGCAGTCAGGCCATGG-BHQ1	Watzinger et al., 2004
15	Epstein Barr virus	BALF5	CGGAAGCCCTCTGGACTTC CCCTGTTTATCCGATGGAATG FAM-TGTACACGCACGAGAAATGCGCC-BHQ1	Kimura et al., 1999
16	Varicella zoster virus	ORF38	AAGTTCCCCCCGTTCGC TGGACTTGAAGATGAACTTAATGAAGC FAM-CCGCAACAACTGCAGTATATATCGTCTCA-BHQ1	Watzinger et al., 2004
17	Herpes simplex 1	gD	CGGCCGTGTGACACTATCG CTCGTAAAATGGCCCCTCC FAM-CCATACCGACCACACCGACGAACC-BHQ1	Weidmann et al., 2003
	Herpes simplex 2	gD	CGCCAAATACGCCTTAGCA GAAGGTTCTTCCCGCGAAAT VIC-CTCGCTTAAGATGGCCGATCCCAATC-BHQ1	Watzinger et al., 2004
18	Herpes virus 6	DNA Pol	TGCTCGGACTGCATCTTGGA TTATTGCCGTGTGTTGCGATT FAM-TTAACATAATCCACCGTGGAACAAAGCATCT-BHQ1	Reddy and Manna, 2005
19	Endogenous retrovirus 3	ENV	CATGGGAAGCAAGGGAACTAATG CCCAGCGAGCAATACAGAATTT FAM-TCTTCCCTCGAACCTGCACCATCAAGTCA-BHQ1	Yuan et al., 2001

“+” *indicates a locked nucleic acid (e.g., +A is a locked nucleic adenine analogue)*.

**Table 2:** Target gene, probe and primer details for RNA viruses.

**Reaction mix**	**Virus**	**Target gene**	**Primer, probe sequences (5^′^-3^′^)**	**Source**
1	Rhinovirus	5′ UTR	CY+AGCC+TGCGTGGY GAAACACGGACACCCAAAGTA FAM-TCCTCCGGCCCCTGAATGYGGC-BHQ1	Arden and Mackay, 2010
2	Influenza A	Matrix	CTTCTAACCGAGGTCGAAACGTA GGTGACAGGATTGGTCTTGTCTTTA Q670-TCAGGCCCCCTCAAAGCCGAG-BHQ2	Whiley and Sloots, 2005
3	Influenza B	Matrix	GCATCTTTTGTTTTTTATCCATTCC CACAATTGCCTACCTGCTTTCA FAM-TGCTAGTTCTGCTTTGCCTTCTCCATCTTCT-BHQ1	Lambert et al., 2008
	Influenza C	Matrix	CATAATTGAACTTGTCAATGGTTTTGT TTCAGGCATAATTGTGGTCTTTATATCT FAM-CTCGGCAGATGGGAGAGATGGTGTG-BHQ1	Personal communication
4	RSV A	Nucleocapsid	AGATCAACTTCTGTCATCCAGCAA TTCTGCACATCATAATTAGGAGTATCAAT FAM-CACCATCCAACGGAGCACAGGAGAT-BHQ1	Van Elden et al., 2003
	RSV B	Nucleocapsid	AAGATGCAAATCATAAATTCACAGGA TGATATCCAGCATCTTTAAGTATCTTTATAGTG YAK-TATGTCC+AGG+TTAGGAAG+G+G+AA-BBQ	
5	Parainfluenza 1	Hemagglutinin-neuraminidase	TTTAAACCCGGTAATTTCTCATACCT CCCCTTGTTCCTGCAGCTATT FAM-TGACATCAACGACAACAGGAAATCATGTTCTG-BHQ1	Lambert et al., 2008
	Parainfluenza 2	Nucleocapsid	AGAGTTCCAACATTCAATGAATCAGT CTCAAGAGAAATGTCATTCCCATCT YAK-CCTCTGTATTGCTCATGCATAGCACGGA-BBQ	
6	Parainfluenza 3	Nucleocapsid	CGGTGACACAGTGGATCAGATT AGGTCATTTCTGCTAGTATTCATTGTTATT Q670-TCAATCATGCGGTCTCAACAGAGCTTG-BHQ2	
	Parainfluenza 4A	Phosphoprotein	GCAATTAAGGCAYTAGAAGTRA AATTGTGGCAAGTGAACC FAM-TTTGTCAACTTTCCCYTCAATCCTG-BHQ1	Wang et al., 2012
	Parainfluenza 4B	Phosphoprotein	TCCHATAATCGTCACTGGYA TATTTTAAGTGCATCTATACGAAC Q670-ACAAAATGGGTCTTGCTARCGG-BHQ2	
7	Coronavirus HKU1	Polymerase	CCTTGCGAATGAATGTGCT TTGCATCACCACTGCTAGTACCAC FAM-TGTGTGGCGGTTGCTATTATGTTAAGCCTG-BHQ1	Dare et al., 2007
8	Coronavirus OC43	Nucleocapsid	CGATGAGGCTATTCCGACTAGGT CCTTCCTGAGCCTTCAATATAGTAACC Q670-TCCGCCTGGCACGGTACTCCCT-BHQ2	Van Elden et al., 2004
	Coronavirus NL63	Polyprotein 1a	ACGTACTTCTATTATGAAGCATGATATTAA AGCAGATCTAATGTTATACTTAAAACTACG YAK-ATTGCCAAGGCTCCTAAACGTACAGGTGTT-BBQ	Gunson et al., 2005
	Coronavirus 229E	Nucleocapsid	CAGTCAAATGGGCTGATGCA AAAGGGCTATAAAGAGAATAAGGTATTCT FAM-CCCTGACGACCACGTTGTGGTTCA-BHQ1	Van Elden et al., 2004
9	Metapneumo-virus	Nucleocapsid	CATATAAGCATGCTATATTAAAAGAGTCTC CCTATTTCTGCAGCATATTTGTAATCAG FAM-TGYAATGATGAGGGTGTCACTGCGGTTG-BHQ1	Maertzdorf et al., 2004
10	Enterovirus	5′ UTR	CCTGAATGCGGCTAATCC TTGTCACCATWAGCAGYCA FAM-CCGACTACTTTGGGWGTCCGTGT-BHQ1	Oberste et al., 2010

“+” *indicates a locked nucleic acid (eg. +A is a locked nucleic adenine analogue)*.

### Statistical analysis

Statistics were performed using software from Scientific Python, namely SciPy and pandas through the Jupyter Notebook interface (Oliphant, 2007). McNemar's test was used to test for significantly different proportions of viral positivity between sites. Paired Student's t-test was used to compare the mean number of viruses detected between sites. Percentage agreement was calculated for viral detection between both sites for both number and species of viruses detected. Chi square test was used to investigate any correlation between viral presence and control/disease status. Statistical significance was defined as a p-value of < 0.05.

## Results

### Patient characteristics

Twenty-four patients were recruited at time of endoscopic surgery; this included 14 men and 10 women, with an age range of 19–79 years, and a mean age of 51 years. Seven patients had CRS without polyps (CRSsNP), eight had CRS with polyps (CRSwNP), and nine were controls. Demographics and patient characteristics are summarised in Table 3. All patients in the CRS groups underwent functional endoscopic sinus surgery (FESS), while those in the control group underwent trans-sphenoidal resections of pituitary masses.

**Table 3:** Summary of patient demographics and characteristics.

**Mean age (years)**	**46.5**	**45.6**	**61.1**
Sex	3 M, 6 F	4 M, 3 F	7 M, 1 F
Diagnosis	9 controls	7 CRSsNP	8 CRSwNP

### Viral detection and analysis

ERV3 was detected in all patient samples, with a median Ct of 22.5 (range 19.3–28.0), showing adequate cellular material was captured throughout the collection and DNA extraction phases. Eighteen patients were positive for at least one virus in at least one site (18/24, 75%), while six (6/24, 25%) were negative for any of the viruses for which the samples were screened (Table 4). Similar rates of viral detection were seen between the MM and IM overall (52% positivity at the MM vs. 48% at the IM; p = 0.55, McNemar's test). The mean number of viruses detected at the MM was 0.875 ± 0.899, vs. 0.750 ± 1.032 at the IM. The mean number of viruses detected did not differ significantly between both sites (p = 0.57, paired t-test). Interestingly the majority of patients (63%) did not show an intranasal correlation between sites. Of the nine patients demonstrating similar findings at both sites, only three demonstrated viral presence with six showing an absence of virus at all sites. Fifteen patients were inconsistent between the two sites; this included four patients who exhibited virus or viruses at both sites but of different species at each (Table 4) These findings correspond to a percentage agreement of only 31 between the MM and IM in terms of number of viruses detected (i.e., not accounting for viral species). When analysing for viral species there was only a percentage agreement of 27 between the sites. No correlation was found between control/disease phenotype and viral presence (p = 0.68, Chi-square test).

**Table 4:** Viral species identified at middle and inferior meatuses.

**Patient number**	**Diagnosis**	**Site of sampling**	**Viruses identified**	**Patient number**	**Diagnosis**	**Site of sampling**	**Viruses identified**
1	Control	MM	None	13	Control	MM	EBV, HHV6
		IM	None			IM	EBV
2	CRSwNP	MM	None	14	CRSsNP	MM	None
		IM	None			IM	Influenza A, HHV6
3	CRSsNP	MM	None	15	CRSsNP	MM	None
		IM	None			IM	EBV
4	Control	MM	None	16	CRSwNP	MM	EBV
		IM	None			IM	None
5	CRSwNP	MM	None	17	CRSsNP	MM	HHV6
		IM	None			IM	None
6	Control	MM	None	18	Control	MM	HHV6
		IM	None			IM	None
7	CRSwNP	MM	Influenza A, HHV6	19	CRSwNP	MM	None
		IM	Influenza A, HHV6			IM	EBV
8	Control	MM	HHV6	20	CRSwNP	MM	EBV, HHV6
		IM	HHV6			IM	None
9	Control	MM	EBV, HHV6	21	CRSsNP	MM	HHV6
		IM	EBV, HHV6			IM	None
10	CRSsNP	MM	HHV6	22	CRSwNP	MM	EBV, HHV6
		IM	EBV			IM	None
11	CRSsNP	MM	Influenza A	23	CRSwNP	MM	PIV2
		IM	Influenza A, EBV			IM	None
12	Control	MM	HHV6	24	Control	MM	None
		IM	EBV			IM	EBV

## Discussion

A standardised, validated technique for viral sampling in the sinonasal passages has not yet been described. This study shows a significant discrepancy in viral presence and species between just two of the sites commonly sampled, highlighting the need for such a standardisation. This indicates that viral sampling needs to be conducted with a cytobrush in both the IM and MM.

Collection variability has the potential to impact respiratory viral detection significantly. The sample volume and location, as well as the documented uneven distribution of viruses within the nasal cavity, can all contribute to false negatives (Van Wesenbeeck et al., 2014). Given that clinically relevant, actively replicating viruses of the upper respiratory tract are intra-cellular and largely reside in the upper epithelial layers of the mucosa (Vareille et al., 2011), adequate cell sampling is an important consideration when searching for viruses. Traditional viral sampling brushes have the advantages of causing less trauma to the delicate mucosa and thus less discomfort to a conscious patient, but risk sampling largely secreted elements rather than the cells themselves (Spyridaki et al., 2009). Viruses do certainly reside in these secretions, but this may not necessarily represent actively replicating virus causing disease. For these reasons we elected to use cytology brushes for this study. Cytology brushes are designed specifically for cell sampling due to their larger and more rigid design than traditional viral sampling brushes. Although this may potentially increase the risk of trauma or discomfort to the awake patient, when used in the anaesthetised patient, as was the case in this study, they have the significant advantage of increased cellular sampling yield (Stokes et al., 2014).

As mentioned viral yields are also difficult to compare in respiratory samples, as sample volume can vary widely. The samples here averaged a DNA concentration of 96.37 ng/μL and an RNA concentration of 58.58 ng/μL, but with ranges of 10.3–383.3 and 4–247.3 ng/μL, respectively. To minimise the impact of this variability on results all samples underwent an ERV3 assay prior to PCR. This has been identified previously as a positive indicator of respiratory sample quality, and all samples were well-within previously published target ranges (Alsaleh et al., 2014; Sarna et al., 2017).

Viral sampling is traditionally performed from the inferior nasal septum and anterior nasal floor as they are easily accessible and cause minimal patient discomfort. The posterior nasopharyngeal wall is also traditionally endorsed, but confirmation of access to this site is difficult without endoscopic equipment. There is no evidence however that these three sites are any more or less appropriate. These areas may indeed be less than ideal due to their relative proximity to airborne pathogens (and therefore risk of contamination), their distance from areas of particular interest (such as the paranasal sinuses), and the tendency for pooling of potentially contaminating secretions in these areas. The MM (sampled in our study) remains relatively simple to access but is further away from potential sources of contamination, and receives drainage from a much wider area including the maxillary, frontal, and anterior ethmoid sinuses. There are indeed a number of other sites in the nasopharynx not investigated here, for example the superior meatus, the sphenoethmoidal recess, and the post-nasal space, however these are difficult to reach without endoscopic equipment that is not readily available in the primary care setting, and can be subject to contamination from other more anterior sites during insertion and removal of sampling instruments. Should these areas demonstrate greater viral presence than the MM and IM the specialist input required to access the sinuses themselves would likely delay or miss altogether the diagnosis and window for anti-viral treatment. Large-scale economic viability of the collection method here proposed also warrants mention; pooling of viral samples from the same patient prior to analysis and limitation of viral testing to a smaller panel of more prevalent, clinically relevant pathogens would be prudent, however selection of such a panel requires further investigation.

Common, clinically relevant upper respiratory viruses are largely of the RNA subtype, and include RV, influenza, RSV, and hMPV, and to a lesser extent CoV, PIV, and EnV. Of the DNA viruses here investigated AdV is certainly a notable URT pathogen. BoV has been linked largely with lower respiratory illness (Gottlieb, 2018). The other DNA viruses here investigated were chosen not primarily for their clinical relevance in viral respiratory disease, but instead for either their near-ubiquity, their ability to remain latent in the respiratory tract, or both. EBV and HHV6 have here shown themselves to be particularly useful in testing viral sampling methods as they are almost omnipresent in the adult sinonasal passages, and are rarely entirely cleared after first infection.

Effort was made in this study to identify any correlation between control/CRSsNP/CRSwNP status and viral presence. Patient reports of recent viral infection, sinonasal outcome test 22 (SNOT-22) scores, Lund-Mackay computed tomography scores, Lund-Kennedy endoscopic scores, and RT-PCR cycle threshold values were collected for all patient and samples, but the sample size here was too small to demonstrate any significant differences. The inclusion of the extremely common herpesviruses (seen, as expected, in many of our controls) also skewed any such results. This is an area that requires significant further investigation.

Neither of the sites from whence samples were taken were more or less likely to be positive than the other. Our observation that the MM and IM only completely agree in terms of viral presence or absence as well as viral species in 27% of cases indicates a significant proportion of viruses present would not be identified were only one site to be sampled. Our findings suggest viral sampling from the sinonasal passages should be taken from both sites in both nasal cavities. The sampling method here described has significant implications for further research into a field of emerging importance in both rhinologic and also respiratory disease on a larger scale.

## Data availability statement

The raw data supporting the conclusions of this manuscript will be made available by the authors, without undue reservation, to any qualified researcher.

## Author contributions

RG contributed to study design, sample collection and processing, data analysis, and writing of the manuscript. CB contributed to sample collection. AB contributed to statistical analysis. SB contributed to sample processing and writing of the manuscript. SV contributed to study design and review of the manuscript. P-JW and AP contributed to study design, sample collection and review of the manuscript.

### Conflict of interest statement

The authors declare that the research was conducted in the absence of any commercial or financial relationships that could be construed as a potential conflict of interest.
